# Prevalence and Predictors of Urinary Tract Infections among Children with Cerebral Palsy in Makurdi, Nigeria

**DOI:** 10.1155/2013/937268

**Published:** 2013-11-25

**Authors:** Emmanuel Adémólá Anígilájé, Terkaa Terrumun Bitto

**Affiliations:** ^1^Department of Paediatrics, Benue State University, P.M.B. 102119, Makurdi, Nigeria; ^2^Department of Epidemiology and Community Health, Benue State University, P.M.B. 102119, Makurdi, Nigeria

## Abstract

*Background*. Children with cerebral palsy (CP) are prone to urinary tract infection (UTI). *Methods/Objectives.* The prevalence and the predictors of UTI among children with CP were compared to age- and sex-matched children without CP at Federal Medical Centre, Makurdi, Nigeria, from December 2011 to May 2013. *Results*. The age range was between 2 and 15 years with a mean age of 8.63 ± 3.83 years including 30 males and 22 females. UTI was confirmed in 20 (38.5%) CP children compared to 2 children (3.8%) without CP (*P* value 0.000). Among CP children, *Escherichia coli* was the commonest organism isolated in 9 (9/20, 45.0%), *Streptococcus faecalis* in 4 (20.0%), and *Staphylococcus aureus* in 3 (15%), while both *Proteus spp.* and *Klebsiella spp.* were isolated in 2 children (10.0%) each. *Escherichia coli* was also found in the 2 children without CP. All the organisms were resistant to cotrimoxazole, nalidixic acid, nitrofurantoin, and amoxiclav, while they were 100% sensitive to ceftriaxone and the quinolones. In a univariate regression analysis, only moderate to severe gross motor dysfunction predicted the risk of UTI (OR = 54.81, 95% CI, 2.27–1324.00, *P* value 0.014). *Conclusion*. Efforts should be put in place to aid mobility among CP children in order to reduce the risk of UTI.

## 1. Introduction

A child with cerebral palsy is having a difficulty in neuromotor control, a nonprogressive brain lesion, and an injury to the brain that occurred before it was fully matured [1]. Cerebral palsy is a common cause of childhood morbidity [1]. This morbidity comprised seizure disorders, mental retardation, abnormalities of vision, problems with respiratory muscle, and lower urinary tract dysfunctions. [1] The lower urinary tract dysfunctions are manifested symptomatically as urinary incontinence, urgency, frequency, hesitancy, and urinary tract infection [2]. Possible reasons for the propensity to urinary tract infections include vesicoureteral reflux and incomplete bladder emptying resulting from detrusor hyperreflexia and detrusor sphincter dyssynergia [2–7]. In addition, the impaired cognition and the inability to communicate bladder fullness and the need to void, together with an impaired mobility, may also explain the tendency to urinary retention and the attendant risk of urinary tract infections [2, 8]. A prevalence of 2.2–32.5% of urinary tract infections among cerebral palsy patients has been reported by authors from developed countries [2, 9, 10]. Unfortunately, there has been no report of UTI among patients with cerebral palsy in Nigeria. This study therefore aims at determining the prevalence and predictors of UTI among cerebral palsy patients attending the Paediatric Outpatient Department of a tertiary health facility in Makurdi, Northern Nigeria. This study becomes important because of the potential complications that can result from missed and untreated urinary tract infections in children. 

## 2. Materials and Methods

Ethical approval for the study was obtained from the Hospital Research and Ethics Committee. Children were recruited into the study if the parents or caregivers had given their consent. Subjects were consecutive patients with cerebral palsy attending the Neurologic Clinic of the Paediatrics Outpatient Department (POD) of the Federal Medical Centre, Makurdi. Controls were age (to the nearest birthday) and sex matched consecutive children, who were also attending follow-up appointments at the POD having been previously admitted, treated, and had recovered from ailments such as severe malaria, lower respiratory tract infections, and diarrheal disease. Subjects and controls that had taken antibiotics in the preceding two weeks, those with on-going menses or one who is having vaginal/penile discharge, were excluded from the study. Recruitment into the study spanned between December 2011 and May 2013. The clinical, topographical, and gross motor dysfunctions and Intelligent Quotients characterizations of the subjects were done by the principal author. The gross motor function classification system (GMFCS) as previously described was employed to classify the severity of functional impairment in children with cerebral palsy [11]. GMFCS is a 5-level system defined by separating 4 age groups. It emphasizes sitting and walking functions of children, focusing on self-initiation of the action. Patients with GMFCS level 1-2 were classified as mild, those with level 3 were classified as moderate, and those with level 4-5 were classified as severe CP. The intellectual state of the subjects with cerebral palsy could only be tested using the Man-Drawing Quotient by Ziler that has been previously validated by Ebigbo and Izuora among Nigerian children aged 6 to 11 years [12]. Children with Intelligent Quotient ≤ 75% were considered to be intellectually disabled. A well structured pretested questionnaire was employed to obtain information from the parents or the caregivers—of the subjects and the controls—regarding other demographic data, history suggestive of urinary tract infection, enuresis, and constipation. Symptoms of urinary tract infections captured include fever, painful urination (dysuria; either verbally communicated to the mother of the child or mother noticed persistent crying on micturition), frequent micturition (frequency of urination of more than 7 times a day), gross haematuria, cloudy or smelly urine, and flank/back pain. Constipation was as defined by the Paris consensus on childhood constipation terminology (PACCT) [13] “a period of 8 weeks with at least 2 of the following symptoms: defecation frequency less than 3 times per week, fecal incontinence frequency greater than once per week, passage of large stools that clog the toilet, palpable abdominal or rectal fecal mass, stool withholding behavior, or painful defecation.” Enuresis was defined according to the fourth edition of the Diagnostic and Statistical Manual of Mental Disorders [14] (DSM-IV) as involuntary or unintentional repeated voiding of urine, into bed or clothes, which occurs twice a week for at least 3 consecutive months, in a child that is at least 4 years old. Parents or caregivers of subjects and the control were provided with a properly labeled uncontaminated universal bottle for the collection of midstream early morning urine to be brought back when coming for their next clinic visit. The parents or the caregiver was also instructed on how to collect the midstream urine. Routine personal hygiene is all that was required. When test cannot be done within the first hour of urine collection, urine was stored in the refrigerator (at 4°C) and tested within 4 hours of storage in the refrigerator. Incubation of the urine was carried out on sheep blood agar, McConkey or CLED (cysteine lactose electrolyte deficient) agar using the quantitative loop method. The plates were examined at between 18 and 24 hours. A yield of multiple organisms was considered as a contaminant. Sensitivity of organisms was done using the is osensitive tested agar plate and incubated at 37°C for 18–24 hours. The zones of inhibition greater than 15 mm were regarded as sensitive. A urine culture was repeated if there was a contaminant yield. Urinalysis was done using Multistix 10SG by BAYER DIAGNOSTIC and urine sediment microscopy in the standard method [15]. Urinalyses were analysed for significant proteinuria, significant haematuria, the presence of nitrite, significant pyuria, and significant microscopic pyuria. Radiological investigations including abdominal ultrasound scan (USS) and micturating cystourethrogram (MCUG) were carried out among subjects and control with confirmed UTI.

For the purpose of this study, the following definitions were applied:* Asymptomatic bacteriuria (ASB) or asymptomatic urinary tract infection (AUTI)* is defined as the quantitative growth of bacteria, greater than or equal to 10^5^ colony forming units per millilitre urine of the same organism, on collected midstream urine specimens, in the absence of symptoms of urinary tract infection. 

*Symptomatic bacteriuria (SB) or symptomatic urinary tract infection (SUTI)* is defined as the quantitative growth of bacteria, greater than or equal to 10^5^ colony forming units per millilitre urine of the same organism, on collected midstream urine specimens, in the presence of one or more than one of these signs and, or symptoms of urinary tract infection including fever, dysuria, gross haematuria, cloudy or smelly urine, frequency of urination, and flank/back pain.

 
*Significant proteinuria* is a presence of 2+ or more protein in the urine.
 
*Significant haematuria* is a presence of 2+ or more blood in the urine.
 
*Significant pyuria* is a presence of 2+ or more leucocyte in the urine.
 
*Significant microscopic pyuria* is white blood cell (WBC) count of 10 and above per high power field (HPF).

### 2.1. Data Analysis

The statistical analysis was done using SPSS version 16. Test between dependent and independent variables was carried out using the Chi-squared test (*χ*2). The logistic regression model was used to determine independent predictors (demographic, signs, and symptoms) of urinary tract infection. Only independent variables with *P* value of <0.1 at the bivariate analysis were considered for logistic regression. Odd ratios with 95% confidence intervals were used to measure the strength of the association at the statistical significance level of *P* < 0.05.

## 3. Results

A total of 93 children with cerebral palsy were seen within the study period but only 52 were included in the study. Twenty children did not meet the inclusion criteria. Fourteen children were lost to follow-up, and they could not return the urine specimens. Mothers were unable to obtain urine specimens in 7 children. The age range was between 2 and 15 years with a mean age of 8.63 ± 3.83 years including 30 males and 22 females with a male to female ratio of 1 : 0.7. With respect to the types of cerebral palsy, 19 (36.5%) had spastic hemiplegia, 16 (30.8%) had spastic diplegia, 11 (21.2%) had spastic quadriplegia, 4 (7.7%) had a mixed combination of athetosis with spastic hemiplegia, and another 2 (3.8%) children with hydrocephalus had ataxic cerebral palsy. Regarding the degree of gross motor dysfunction, 30 (57.7%) had mild dysfunction including 11 (21.2%) with grade I and 19 (36.5%) with grade II. Moderate to severe motor dysfunction is seen in 22 (42.3%) including eight (15.4%) with grade 3, five (9.6%) with grade 4, and nine (17.3%) with grade 5. Among the 34 children with CP whose ages were between 6 and 11 years and for which Ziler's Man-Drawing Quotient was determined, 9 had scores ≤ 75% (i.e. Intellectually disabled) and 15 had scores above 75% (Table 1).

### 3.1. Urinary Symptoms/Signs: Constipation and Urinalyses Findings among Subjects with Cerebral Palsy and Controls without Cerebral Palsy

Table 2 shows that the symptoms and signs of UTI, a history of constipation, enuresis, a prior history of UTI, urinalyses findings including significant haematuria, significant proteinuria, nitrite, significant pyuria, significant microscopic pyuria, and culture proven UTI were significantly more frequent in subjects with cerebral palsy than their age and sex-matched comparators without cerebral palsy (*P* value <0.05).

### 3.2. Urinary Culture and Sensitivity Patterns among Subjects with Cerebral Palsy and Controls without Cerebral Palsy

Table 3 shows that urinary tract infection was confirmed in 20 (38.5%) over-five children with cerebral palsy comprising twelve (23.1%) males and eight (15.4%) females with a male to female ratio of 1.5 : 1 (*P* value 0.790). Asymptomatic urinary tract infection (AUTI) was seen in 2 (10.0%) children and symptomatic urinary tract infection (SUTI) in the remaining 18 (90.0%) of cerebral palsy children. SUTI was confirmed in 2 controls children without CP.

*Escherichia coli* were isolated from majority of subjects, 9 (9/20, 45.0%), *Streptococcus faecalis* in 4 (20.0%), and *Staphylococcus aureus* in 3 (15%), while both *Proteus spp*. and *Klebsiella spp*. were isolated in 2 children (10.0%) each. All the three children with *Staphylococcus aureus* isolates also presented with fever.

The major isolates, *Escherichia coli*, were 100% sensitive to ciprofloxacin, ofloxacin, sparfloxacin and ceftriazone but less sensitive to gentamycin (50%–66.7%) and were resistant to nitrofuratoin (0%), tetracycline (0%), cotrimoxazole (0%), amoxiclav (0%), and nalidixic acid (0%). *Streptococcus faecalis*, *Staphylococcus aureus*, *Proteus spp*. and *Klebsiella spp*. were also 100% sensitive to the quinolones and ceftriazone and were also resistant to nitrofuratoin, tetracycline, cotrimoxazole, amoxiclav, and nalidixic acid but were between 33.3%–50% sensitive to streptomycin and between 33.3%–75% sensitive to gentamycin.

### 3.3. Abdominal Ultrasound Finding

Abdominal ultrasound scan among the 20 CP children with UTI revealed no renal parenchymal abnormality. In 2 female children there were bladder wall thickness, irregularity of the bladder wall, and residual urine. Vesicouretera reflux (VUR) was seen in only one of the two female children on micturating cystourethrogram. Radiological findings were normal in the two control children without CP. Urodynamic study was not done and causal relationship between the VUR and bladder dysfunction could not be established.

### 3.4. Predictors of Urinary Tract Infections

In bivariate analysis, older children (>5 years), moderate to severe motor disability, dysuria, constipation, flank/abdominal pain, significant haematuria, significant pyuria and significant microscopic pyuria, were found to be significantly associated with UTI (*P* values <0.05). However, in univariate regression analysis only moderate to severe gross motor dysfunction predicts the risk of UTI (OR = 54.81, 95% CI, 2.27–1324.00, *P* value 0.014). Multivariate regression analysis could not be done (Tables 4, 5 and 6).

## 4. Discussion

To the best of our knowledge this is the first study describing the burden of urinary tract infection among Nigerian children with CP and the prevalence of 38.5% found in the present study was higher than those documented among other Nigerian children at risk of UTI including those with sickle cell anaemia (5.8–21.6%), [16–18] malaria (6–9%), [19, 20], HIV (10.3%) [21], and malnutrition (11–11.3%) [22, 23].

As may be expected, the frequency of UTI in the children with CP (38.5%) in our study was higher than their comparators without CP (3.8%) and the 1–3% reported in the general paediatric population [24]. The possible reasons for the higher prevalence of UTI among CP children had been enumerated earlier [2–9]. 

Furthermore, the frequency of UTI of 38.5% found in the present study is comparable to the 32.5% reported by Ozturk et al. in Turkey [10], but is much higher than the respective 7.4% and 2.2% reported by Reid and Borzyskowski in London [2] and Hellquist et al. in North Carolina [9]. Unlike the present study, antibiotics had been used prior to presentation of the CP patients in the latter two studies [2, 9] and may possibly explain the discrepancies in the frequency of the UTI. 

Furthermore, the symptoms and signs of UTI, a history of constipation, enuresis, a prior history of UTI, urinalyses findings, and culture proven UTI were significantly more frequent in subjects with cerebral palsy than their age- and sex-matched comparators without cerebral palsy. Similar findings have also been reported by Ozturk et al. in Turkey [10]. 

In univariate regression analysis, only moderate to severe gross motor dysfunction significantly predict the risk of UTI among our subjects with CP. These are children who often have to be carried from one place to the other by their siblings or their parents because of difficulty in mobility and both manually propelled or electrically powered wheelchairs are often beyond the reach of these families. Often, these children are neglected and, stationed supine in one place for a long time, with the majority developing pressure sores on the occiputs and the buttocks and the poor personal hygiene resulting from prolonged smearing by their faeces may increase the risk of UTI. Also, because of poor mobility, UTI may develop easily following urinary retention resulting from the difficulty in getting to the toilets to micturate in a few that may be urinary continent. Closely linked to the poor mobility is the poor water intake and the resultant risk of kidney stones which may predispose these children to UTI [25]. In addition, a high burden of pinworms seen in some of these children [26] may be linked to a higher risk of UTI. Furthermore, the propensity to developing constipation in poorly mobile CP children may also have contributed to the higher risk of UTI in this group of children. 

In the present study, we did not find any association between intellectual disability and UTI. We propose that cognitive ability to communicate the need to void as well as its effect on urinary continence attainment may not be as important as improving mobility among our CP children in order to reduce the prevalence of UTI.

In addition, we found that all the CP subjects with UTI are over-five children. These findings may probably result from recruitment bias as more CP patients that are over five (75%) were recruited in our study. 

Furthermore, UTI presents more symptomatically -18/20, 90% in our CP patients, and therefore all efforts should be put in place to review symptoms of UTI among the CP patients when they come for follow-up in our clinics in order to confirm and treat a UTI and thus prevent its potential complications. 

*Escherichia coli* have been shown to account for up to 75% of UTIs in all paediatric age groups followed by *Klebsiella spp*., *Proteus spp*., and *Pseudomonas spp*. [27]. As may be expected, *Escherichia coli* were also the commonest isolates (9/20, 45%) among our subjects with CP and the controls without CP (2/2, 100%) in the present study. 

Previous studies [17–20, 23] and the present one had shown that *Escherichia coli* is becoming highly resistant to the first line empirical antimicrobials for UTI, including cotrimoxazole, amoxicillin, nitrofuratoin and nalidixic acid. Its preserved sensitivity to the quinolones and ceftriazone and gentamycin may be explained by the fact that the quinolones are rarely prescribed for children and the parenteral routes of ceftriazone and gentamycin reduce the abuse of these two antibiotics. Although in vitro resistance may not necessarily means in vivo resistance, Chukwu et al. [17] had earlier summarized some of the common reasons for the emergence of resistance to the first line antimicrobials. These include a mix of drug resistance developed by the pathogens, low patient compliance, self-medication, and potentially sub-therapeutic prescription by health workers [17]. The menace of substandard antibiotics which is common in developing countries cannot also be overemphasized.

We were able to detect VUR in one subject with CP. The causal relationship between this VUR and the possible neuropathic bladder of CP could not be ascertained because urodynamic studies cannot be done in our centre.

*Limitation of the Study.* A larger population of children with CP could have made the prevalence of UTI as found in the present study more representative in this group of children. 

Urodynamic study was not performed on the subjects studied and so the exact judgment about predisposing lesions could not be ascertained.

Intelligence Quotients were also only tested among CP children between the ages of 6–11 years because other evaluation methods are yet to be validated and standardized for use in Nigerian children.

## 5. Conclusion

The present study would sum up to indicate that there is a high prevalence of UTI among our children with CP, especially among those with severe immobility. Therefore, concerted efforts be put in place for effective physiotherapy aimed at attaining the greatest possible mobility and independence among our children with CP.

## Figures and Tables

**Table 1:** Some demographic characteristics, GMFCS, and Intelligent Quotients of subjects with CP.

Characteristics	No.	Percentage (%)
Age		
≤5 years	13	25.0
>5 years	39	75.0
Total	**52**	**100.0**

Gender		
Female	22	42.3
Male	30	57.7
Total	**52**	**100.0**

Types of CP		
Spastic hemiplegia	19	36.5
Spastic diplegia	16	30.8
Spastic quadriplegia	11	21.2
Ataxic CP	02	3.8
Mixed	04	7.7
Total	**52**	**100.0**

Gross motor dysfunction		
1	11	21.2
2	19	36.5
3	08	15.4
4	05	9.6
5	09	17.3
Total	**52**	**100.0**
Intellectual disability		
No	15	62.5
Yes	9	37.5
Total	**24**	**100.0**

**Table 2:** Urinary symptoms, constipation, and urinalyses findings among subjects with cerebral palsy and controls without cerebral palsy.

Characteristics	Cerebral palsy (*n* = 52)	Children without CP (*n* = 52)	*P* value
Symptoms			
Fever	17 (32.7%)	2 (3.8%)	0.0002
Flank/abdominal pain	17 (32.7%)	— (0%)	0.0000
Frequency of urination	27 (51.9%)	— (0%)	0.0000
Dysuria	8 (15.4%)	1 (1.9%)	0.1600
Constipation	21 (40.4%)	7 (13.5%)	0.0026
Enuresis			
1°	22 (42.3%)	9 (17.3%)	0.0063
2°	4 (7.7%)	— (0%)	0.0439
Prior history of UTI	20 (38.5%)	7 (13.5%)	0.0045

Urinalysis			
Significant haematuria	13 (25.0%)	4 (7.7%)	0.0189
Significant proteinuria	15(28.8%)	6 (11.5%)	0.0301
Significant pyuria	13 (25.0%)	4 (7.7%)	0.0189
Nitrite	12 (23.1%)	1 (1.9%)	0.0015
Urine microscopy			
Significant microscopic pyuria	12 (23.1%)	2 (3.8%)	0.0048
Urine Culture	20 (38.5%)	2 (3.8%)	0.0000

**Table 3:** Urinary culture and sensitivity patterns among subjects with cerebral palsy and controls without cerebral palsy.

	CP					NO CP
	*E. coli *	*Strept. faecalis *	*Staph. aureus *	*Proteus spp. *	*Klebsiella spp. *	*E. coli *
No. of Significant growth	9	4	3	2	2	*2 *

Sensitivity						
Ciprofloxacin	9 (100%)	4 (100%)	3 (100%)	2 (100%)	2 (100%)	*2 (100%) *
Ofloxacin	9 (100%)	4 (100%)	3 (100%)	2 (100%)	2 (100%)	* 2 (100%) *
Sparfloxacin	9 (100%)	4 (100%)	3 (100%)	2 (100%)	2 (100%)	*2 (100%) *
Ceftriazone	9 (100%)	4 (100%)	3 (100%)	2 (100%)	2 (100%)	*2 (100%) *
Gentamycin	6 (66.7%)	4 (75%)	1 (33.3%)	1 (50%)	1 (50%)	*1 (50%) *
Nalidixic acid	0 (0%)	0 (0%)	0 (0%)	0 (0%)	0 (0%)	*0 (0%) *
Streptomycin	5 (55.6%)	2 (50%)	1 (33.3%)	1 (50%)	1 (50%)	*1 (50%) *
Amoxiclav	0 (0%)	0 (0%)	0 (0%)	0 (0%)	0 (0%)	*0 (0%) *
Tetracycline	0 (0%)	0 (0%)	0 (0%)	0 (0%)	0 (0%)	*0 (0%) *
Cotrimoxazole	0 (0%)	0 (0%)	0 (0%)	0 (0%)	0 (0%)	*0 (0%) *
Nitrofuratoin	0 (0%)	0 (0%)	0 (0%)	0 (0%)	0 (0%)	*0 (0%) *

CP: Subjects with cerebral palsy, NO CP: Controls without cerebral palsy, *E. coli*: *Escherichia coli*, *Staph. aureus*: *Staphylococcus aureus*, *Strept. faecalis*: *Streptococcus faecalis*.

**Table 4:** Association between some characteristics of subjects with CP and presence of UTI.

Characteristics	UTI	No UTI	*X* ^2^	*P* value
Age group				
≤5 years	0	13	10.833	0.001
>5 years	20	19		
Total	**20**	**32**		
Gender				
Male	12	18	0.710	0.790
Female	8	14		
Total	**20**	**32**		
GMD				
Mild	02	28	30.286	0.000
Moderate/severe	18	04		
Total	**20**	**32**		
Intellectual disability				
No	6	7	0.906	0.341
Yes	3	8		
Total	**9**	**15**		
Frequency				
No	09	16	0.123	0.726
Yes	11	16		
Total	**20**	**32**		
Dysuria				
No	12	32	15.127	0.000
Yes	08	0		
Total	**20**	**32**		
Constipation				
No	07	24	8.179	0.004
Yes	13	08		
Total	**20**	**32**		
Enuresis				
No	10	16	<0.001	1.000
Yes	10	16		
Total				
Prior history of UTI				
No	11	21	0.587	0.444
Yes	09	11		
Total	**20**	**32**		

**Table 5:** Urinalyses findings among subjects with cerebral palsy and the relationship with UTI.

Characteristics	UTI	No UTI	*X* ^2^	*P* value
Haematuria				
No	10	29	10.833	0.001
Yes	10	03		
Total	**20**	**32**		
Proteinuria				
No	14	23	0.021	0.885
Yes	06	19		
Total	**20**	**32**		
Pyuria				
No	12	27	3.900	0.048
Yes	08	05		
Total	**20**	**32**		
Nitrite				
No	12	28	3.809	0.051*
Yes	08	04		
Total	**20**	**32**		
Microscopic Pyuria				
No	11	29	6.907	0.009*
Yes	09	03		
Total	**20**	**32**		

*Yate's correction was done.

**Table 6:** Predictors of UTI among the children with cerebral palsy.

Variable	UTI		Univariate		
	Positive *n* (%)	Negative *n* (%)	OR	*P* value	95% CI
Age in years					
≤5 years	0 (0.0)	13 (100.0)	NA
>5 years	20 (51.3)	19 (48.7)	
GMD Type					
Mild	2 (6.7)	28 (93.3)	54.81	0.014	2.27–1324.00
Moderate to severe	18 (81.8)	4 (18.2)			
Haematuria					
NO	10 (25.6)	29 (74.4)	53.18	0.188	0.14–19600.00
YES	10 (76.9)	3 (23.1)			
Pyuria					
NO	12 (30.8)	27 (69.2)	2.39	0.714	0.02–250.23
YES	8 (61.5)	5 (38.5)			
Microscopic Pyuria					
NO	11 (27.5)	29 (72.5)	2.00	0.803	0.01–441.62
YES	9 (75.0)	3 (25.0)			
Flank pain					
NO	10 (28.6)	25 (71.4)	3.01	0.562	0.07–125.01
YES	10 (58.8)	7 (41.2)			
Dysuria					
NO	12 (27.3)	32 (72.7)	NA
YES	8 (100.0)	0 (0.0)	
Constipation					
NO	7 (22.6)	24 (77.4)	0.44	0.764	0.002–99.488
YES	13 (61.9)	8 (38.1)			
Nitrite					
NO	12 (30.0)	28 (70.0)	0.05	0.237	0.00–7.37
YES	8 (66.7)	4 (33.3)			

NA: Not available.

## References

[1] Taft LT (1995). Cerebral palsy. *Pediatrics in Review*.

[2] Reid CJD, Borzyskowski M (1993). Lower urinary tract dysfunction in cerebral palsy. *Archives of Disease in Childhood*.

[3] Drigo P, Seren F, Artibani W, Laverda AM, Battistella PA, Zacchello G (1988). Neurogenic vesico-urethral dysfunction in children with cerebral palsy. *The Italian Journal of Neurological Sciences*.

[4] Mayo ME (1992). Lower urinary tract dysfunction in cerebral palsy. *Journal of Urology*.

[5] Bross S, Pomer S, Döderlein L (2004). Urodynamic findings in patients with infantile cerebral palsy. *Aktuelle Urologie*.

[6] Bauer SB (2008). Neurogenic bladder: etiology and assessment. *Pediatric Nephrology*.

[7] Gündoğdu G, Kömür M, Avlan D (2013). Relationship of bladder dysfunction with upper urinary tract deterioration in cerebral palsy. *Journal of Pediatric Urology*.

[8] Fahimzad A, Babaied D, Ghoroubi J, Zahed GH, Rafiei T (2013). Common infections among disabled children admitted to hospital. *Archives of Pediatric Infectious Diseases*.

[9] Hellquist JM, McKinney RE, Worley G (1985). Urinary tract infections in cerebral patients. *Pediatric Research*.

[10] Ozturk M, Oktem F, Kisioglu N (2006). Bladder and bowel control in children with cerebral palsy: case-control study. *Croatian Medical Journal*.

[11] Palisano R, Rosenbaum P, Walter S, Russell D, Wood E, Galuppi B (1997). Development and reliability of a system to classify gross motor function in children with cerebral palsy. *Developmental Medicine and Child Neurology*.

[12] Njokanma OF, Nkanginieme KEO, Azubuike JC, Nkanginieme KEO (2007). Growth and development. *Paediatrics and Child Health in a Tropical Region*.

[13] Benninga M, Candy DCA, Catto-Smith AG (2005). The Paris Consensus on Childhood Constipation Terminology (PACCT) Group. *Journal of pediatric gastroenterology and nutrition*.

[14] American Psychiatric Association (1994). *Diagnostic and Statistical Manual of Mental Disorders*.

[15] Cheesebrough M (2002). *Examination of Urine. District Laboratory Practice in Tropical Countries*.

[16] Ajasin MA, Adegbola RA (1998). Asymptomatic bacteriuria in children with sickle cell anaemia. *Nigerian Journal of Paediatrics*.

[17] Chukwu BF, Okafor HU, Ikefuna AN (2011). Asymptomatic bacteriuria in children with sickle cell anemia at the University of Nigeria teaching hospital, Enugu, South East, Nigeria. *Italian Journal of Pediatrics*.

[18] Asinobi AO, Fatunde OJ, Brown BJ, Osinusi K, Fasina NA (2003). Urinary tract infection in febrile children with sickle cell anaemia in Ibadan, Nigeria. *Annals of Tropical Paediatrics*.

[19] Okunola PO, Ibadin MO, Ofovwe GE, Ukoh G (2012). Co-existence of urinary tract infection and malaria among under five years old: a report from Benin city, Nigeria. *Saudi Journal of Kidney Disease and Transplantation*.

[20] Musa-Aisien AS, Ibadin OM, Ukoh G, Akpede GO (2003). Prevalence and antimicrobial sensitivity pattern in urinary tract infection in febrile under-5s at a children’s emergency unit in Nigeria. *Annals of Tropical Paediatrics*.

[21] Iduoriyekemwen NJ, Sadoh WE, Sadoh AE (2012). Asymptomatic bacteriuria in HIV positive Nigerian children. *Journal of Medicine and Biomedical Research*.

[22] Oyedeji GA (1989). The pattern of infections in children with severe protein energy malnutrition. *Nigerian Journal of Paediatrics*.

[23] Rabasa AI, Shattima D (2002). Urinary tract infection in severely malnourished children at the University of Maiduguri Teaching Hospital. *Journal of Tropical Pediatrics*.

[24] Foxman B (2002). Epidemiology of urinary tract infections: Incidence, morbidity, and economic costs. *American Journal of Medicine*.

[25] Alamdaran A, Naseri M (2007). Urinary tract infection and predisposing factors in children. *Iran Journal of Paediatrics*.

[26] Nwaneri DU, Sadoh AE, Ofovwe GE, Ibadin MO (2012). Prevalence of intestinal helminthiasis in children with chronic neurological disorders in Benin city, Nigeria. *Nigerian Journal of Paediatrics*.

[27] Elder JS, Behrman RE, Vaughan VC, Jensen HB (2004). Urinary tract infections. *Nelson Textbook of Paediatrics*.

